# The influence of two functional genetic variants of GRK5 on tau phosphorylation and their association with Alzheimer's disease risk

**DOI:** 10.18632/oncotarget.20283

**Published:** 2017-08-16

**Authors:** Yuan Zhang, Jianghao Zhao, Mingkang Yin, Yujie Cai, Shengyuan Liu, Yan Wang, Xingliang Zhang, Hao Cao, Ting Chen, Pengru Huang, Hui Mai, Zhou Liu, Hua Tao, Bin Zhao, Lili Cui

**Affiliations:** ^1^ Guangdong Key Laboratory of Age-related Cardiac and Cerebral Diseases, Affiliated Hospital of Guangdong Medical University, Zhanjiang, China; ^2^ Institute for Translational Medicine, College of Medicine, Qingdao University, Qingdao, China; ^3^ Department of Chronic Disease, Shenzhen Nanshan Center for Chronic Disease Control, Shenzhen, China; ^4^ Key Laboratory of Biomedical Information Engineering of Ministry of Education, School of Life Science and Technology, Xi'an Jiaotong University, Xi'an, China; ^5^ Department of Pediatrics, Affiliated Hospital of Guangdong Medical University, Zhanjiang, China; ^6^ Departments of Life Science and Biopharmaceutics, Shenyang Pharmaceutical University, Shenyang, China; ^7^ Clinical Research Center, Affiliated Hospital of Guangdong Medical University, Zhanjiang, China

**Keywords:** GRK5, polymorphisms, p-tau, β-amyloid, Alzheimer’s disease

## Abstract

Our work explores the relationship between G protein-coupled receptor kinase-5 (GRK5) single nucleotide polymorphisms and Alzheimer's disease risk. We confirmed that GRK5 translocates from the cellular membrane to the cytosol in the hippocampus of Alzheimer's disease mice and that GRK5 deficiency promotes tau hyperphosphorylation, a hallmark of Alzheimer's disease pathology. Our results indicate that one functional variant, or mutant, of GRK5 (GRK5-Gln41Leu) decreased GRK5 translocation from the membrane to the cytoplasm and reduced tau hyperphosphorylation, whereas, another GRK5 mutant (GRK5-Arg304His) increased GRK5 translocation to the cytoplasm and promoted tau hyperphosphorylation. In addition, case-control studies revealed that GRK5-Gln41Leu is associated with a lower risk of late-onset Alzheimer's disease. Our findings suggest that the GRK5-Gln41Leu mutant may resist tau hyperphosphorylation by promoting GRK5 membrane stability and, in effect, may contribute to lower Alzheimer's disease risk.

## INTRODUCTION

Alzheimer's disease (AD) is a devastating neurodegenerative disorder clinically characterized by the progressive loss of memory and other neurological functions. Influenced by genetic and environmental factors, the mechanisms leading to AD occurrence are complex. Mutations in three genes, amyloid precursor (*APP*), presenilin 1 (*PSEN1*) and presenilin 2 (*PSEN2*), play extremely important roles in Aβ42 metabolism, and these mutations have been identified as risk factors for Early Onset Alzheimer Disease (EOAD) [1, 2]. The ε4 allele of apolipoprotein E (ApoE) has been identified as an important susceptibility gene for Late Onset Alzheimer Disease (LOAD) [3, 4], while many other genes also associated with LOAD susceptibility to varying degrees.

G protein-coupled receptor kinase-5 (GRK5) is one of the seven GRK family members whose primary function is to desensitize G protein-coupled receptors (GPCRs), which is essential for the desensitization and internalization of M2/M4 receptors [5]. Reduced levels of membrane (functional) GRK5 increased soluble β-amyloid *in vitro*, and decreased GRK5 membrane localization occurs *in vivo* as well as in an AD transgenic model and postmortem human AD brain samples [6, 7]. GRK5 dysfunction mediates the deleterious cycle between cholinergic hypofunction and tau hyperphosphorylation, leading to increased β-amyloid deposition in the hippocampus [8, 9]. Moreover, it has been reported that the proteolytic processing of APP is regulated by a variety of GPCRs, including cholinergic, serotoninergic, and glutamatergic receptors [9], suggesting that GRK5 may also play a role in GPCRs mediated APP metabolic pathway.

Recent studies have characterized the significant genetic variants of GRK5 that modify the risk of disease such as heart failure, hypertension, diabetes and Parkinson's disease [10–12]. It has also been reported that functional genetic polymorphisms of GRK5 could influence β-adrenergic signaling [13, 14]. We propose that the functional genetic polymorphisms in the genes coding for GRK5 may be important pharmacogenetic targets that offer opportunities for novel personalized medicine approaches. To the best of our knowledge, the association of GRK5 polymorphisms with pathogenesis and AD risk has not yet been explored. In the present study, we screened the GRK5 gene for the two main genetic variants in coding region of GRK5 (rs2230345 and rs2230349), exploring the possible influence of the SNPs on GRK5 function and AD risk.

## RESULTS

### GRK5 distribution from membrane to cytosol in the hippocampus of aged APP/PS1 transgenic mice

**Figure 1:**
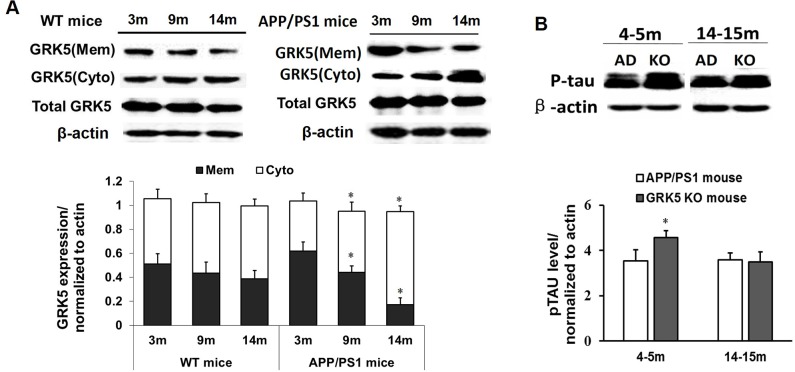
GRK5 translocation from the membrane to the cytosol in the hippocampus of aged APP/PS1 transgenic mice **(A)** Western blotting analysis of GRK5 expression in hippocampal membrane and cytosolic fractions from 3, 9 and 14-month-old APP/PS1 transgenic mice and WT mice. *p<0.05, compared with 3-month-old APP/PS1 transgenic mice. **(B)** Western blotting analysis of p-tau levels in the hippocampus of APP/PS1 transgenic mice and GRK5KO mice at 4-5 months or 14-15 months of age. *p<0.05, compared with APP/PS1 transgenic mice.

The physical location of GRKs, which is determined at least in part by a counterbalance between their binding factors in the membrane and cytosol, is a critical factor regulating interaction, including binding, between GRKs and G-protein-coupled receptors. Thus, we first examined the hippocampi of APP/PS1 transgenic mice of different ages to determine whether the subcellular distribution of GRK5 was altered. As shown in Figure 1A, the GRK5 expression levels in the membrane fraction were significantly decreased in 9-month-old transgenic mice and even more in 14-month-old mice compared with 3-month-old mice and also compare to the WT mice of same age (P<0.05). No significant change was observed in the subcellular fractions between different aged groups of WT mice (Figure 1A).

### GRK5 deficiency induced tau hyperphosphorylation

It has been reported that GRK5 deficiency triggers tau hyperphosphorylation and, thus, causes axonal swelling and functional damage of neurons. To further confirm the effect of GRK5 on tau hyperphosphorylation, we also analyzed p396-tau levels in the hippocampus of APP/PS1 transgenic mice and GRK5KO mice at a range of ages. The level of p-tau in the hippocampus of GRK5KO mice was elevated at 4-5 months aged, which was not significantly different than the APP/PS1 transgenic mice in both young (4-5 months) and older age (14-15 months) groups (Figure 1B). This confirmed that GRK5 deficiency exaggerates tau hyperphosphorylation in the hippocampus *in vivo* in the younger age group. The distribution change of GRK5 also occurs relatively early in the pathological process of AD, and GRK5 deficiency tends to be more susceptible to the effect of Aβ deposition, causing brain dysfunction.

### Selection of GRK5 gene SNPs

**Figure 2:**
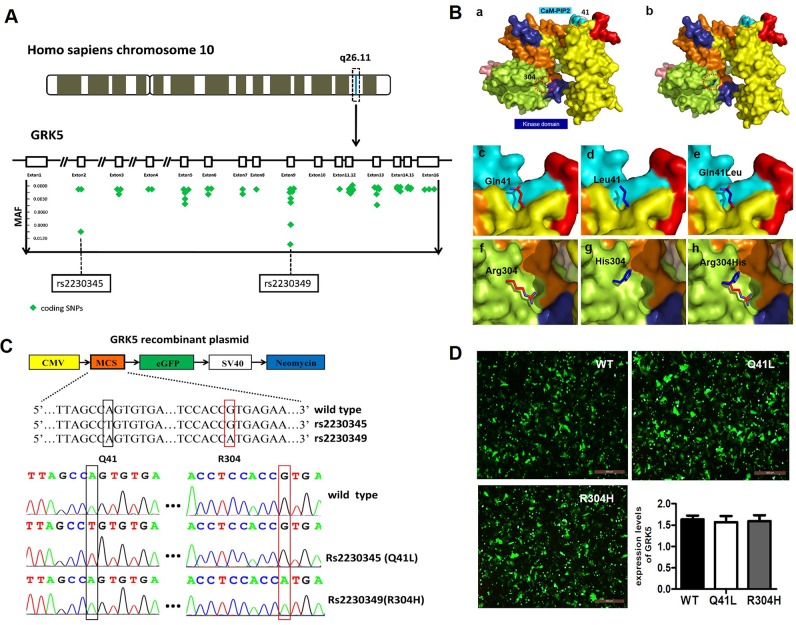
Selection of GRK5 gene SNPs **(A)** Minor allelic frequency of represented SNPs that locate in Homo sapiens GRK5 exons are depicted in green symbols and two sites are eye-catching for their larruping occurrence rate in Chinese Han ethnic group. **(B)** Protein modeling based on rs2230345 (Q41L) and rs2230349 (R304H) GRK5 SNPs. Structural model of the (a) native protein (Q41/R304) and (b) mutant protein (L41/H304) of GRK5. (c)-(e) Superimposed model of native and mutant proteins of GRK5-Q41L. (f)-(h) Superimposed model of native and mutant proteins of GRK5-R304H. Domain organization and important functional regions of GRK5 are represented with different colors, with the C-tail of the kinase domain in indigo blue and the CaM/PIP2-binding sites in cyan. The mutation sites are indicated by red circles. **(C)** Schematic and sequence diagram of two GRK5 mutant vectors, Gln41Leu (rs2230345) and Arg304His (rs2230349). **(D)** SH-SY5Y cells were transfected with Gln41Leu, Arg304His vectors or the empty vector.

The selection of GRK5 SNPs was based on information from databases, such as dbSNP (http://www.ncbi.nlm.nih.gov/SNP/), SNPinfo (http://snpinfo.niehs.nih.gov/) and SNPnexus (http://www.snp-nexus.org/). Two polymorphisms in GRK5 exons with an allele frequency greater than 1% in the Chinese Han ethnic group, GRK5-Gln41Leu (rs2230345, A/T) and GRK5-Arg304His (rs2230349, G/A) variants, were finally selected for further study (Figure 2A). Tertiary structures of WT and two mutant GRK5 proteins encoding the SNPs rs2230345 or rs2230349 were then simulated using comparative modeling to examine the effect of the mutants on GRK5 function, as shown in Figure 2B. The glutamine (Gln) residue at position 41 in the native protein was replaced by leucine (Leu), and the arginine (Arg) residue at position 304 was replaced by histidine (His). The protein structures showed that Gln41 was located in the N-terminal calmodulin/PIP2 binding domain, which is important for GRK5 binding to membranes. The substitution of the polar amino acid Gln41 for the non-polar amino acid Leu41 may enhance GRK5 stability on the membrane, thereby strengthening receptor phosphorylation activity. Arg304 was located in the C-domain, which may interact with the kinase domain, and the substitution of Arg-to-His at amino acid 304 of GRK5 may disrupt the catalytic functions of the kinase domain.

### Influence of GRK5-Gln41Leu and GRK5-Arg304His on GRK5 translocation and tau hyperphosphorylation

**Figure 3:**
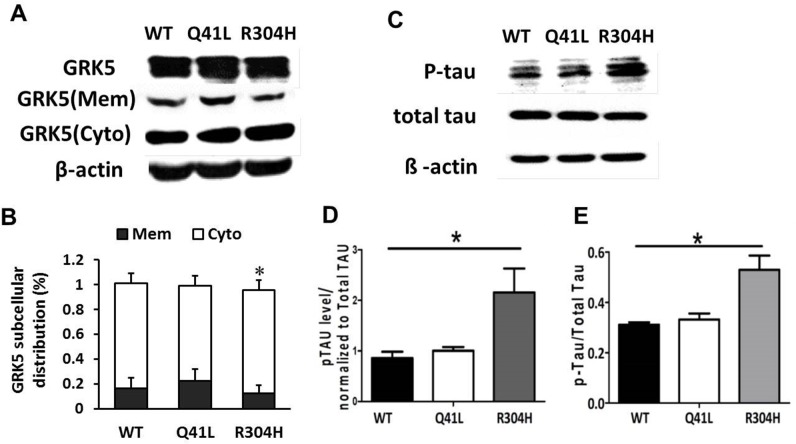
Influence of GRK5-Gln41Leu and Arg304His on GRK5 translocation and tau hyperphosphorylation **(A)** Western blotting analysis of GRK5 levels in GRK5 mutant cells and control cells. **(B)** Quantitative analysis of GRK5 expression in GRK5 mutant cells and control cells. **(C)** Western blotting analysis of p-tau expression in GRK5 mutant cells and control cells. **(D)** Quantitative analysis of GRK5 mutant cells and control cells on p-tau expression. *p<0.05, compared with WT. **(E)** ELISA analysis of p-tau level in GRK5 mutant cells and control cells. *p<0.05, compared with WT.

To explore the possible influence of the Gln41Leu or Arg304His mutations on GRK5 function and tau hyperphosphorylation, the mutant vectors for GRK5-Gln41Leu (rs2230345) and GRK5-Arg304His (rs2230349) were constructed, respectively (Figure 2C). As shown in Figure 2D, SH-SY5Y cells were either transfected with the WT vector or with Gln41Leu or Arg304His mutant vectors with a high efficiency. The expression levels of total GRK5 were not significantly different between the GRK5-Gln41Leu or GRK5-Arg304His groups and the WT group. As shown in Figure 3A and 3B, the level of GRK5 on the membrane in the GRK5-Gln41Leu group was slightly more than the GRK5-Arg304His group, while there was no significant difference compared with the WT group. In the GRK5-Arg304His mutant group, the GRK5 level in the cytoplasm was significantly higher than both the WT and the GRK5-Gln41Leu groups. In addition, western blot and ELISA analyses showed that the level of p-tau was significantly increased in the Arg304His group, and the level of tau phosphorylation in the Gln41Leu group was not significantly different than the WT group (Figure 3C-3E). These results suggest that the GRK5-Arg304His mutant may disrupt GRK5 function, leading to increased expression of p-tau in SH-SY5Y cells.

### GRK5-Gln41Leu rescued tau hyperphosphorylation with low threshold Aβ42 stimulation

**Figure 4:**
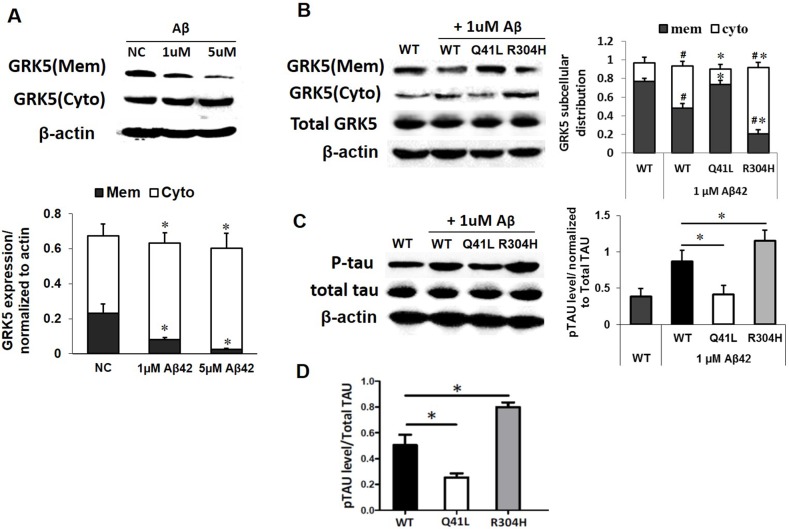
GRK5-Gln41Leu variant protects against low threshold Aβ42 stimulation **(A)** Western blotting analysis of GRK5 expression in membrane and cytosolic fractions of SH-SY5Y cells exposed to 1 μM or 5 μM Aβ42 for 24 h. *p<0.05, compared with NC. **(B)** Western blotting analysis of GRK5 expression in cells with GRK5-Gln41Leu or Arg304His mutations exposed to 1 μM Aβ42.^#^p<0.05, compared with WT; *p<0.05, compared with the group of WT stimulated with Aβ42. **(C)** Western blotting analysis of p-tau in cells with GRK5-Gln41Leu or Arg304His mutations exposed to 1 μM Aβ42. *p<0.05, compared with the group of WT stimulated with Aβ42. **(D)** ELISA analysis of p-tau in cells with GRK5-Gln41Leu or Arg304His mutations exposed to 1 μM Aβ42. *p<0.05, compared with WT.

To determine the impact of β-amyloid on GRK5 function, we exposed SH-SY5Y cells to 1 μM or 5 μM Aβ42 for 24h and separated the cell lysates into membrane and cytosolic fractions for subsequent Western blotting (Figure 4A). Distribution of GRK5 occurred at the 1 μM threshold of Aβ42 oligomer stimulation but not at the 5 μM threshold, indicating a possible dose-dependent mechanism. Therefore, the 1 μM Aβ42 oligomer dose was chosen for use is subsequent experiments.

We found that after low threshold Aβ42 stimulation, the distribution of GRK5 in the Gln41Leu group was clearly different than the WT group. However, there was no significant difference between the WT group and the GRK5-Gln41Leu with Aβ42 stimulation group. Whereas, GRK5 levels in the GRK5-Arg304His with Aβ42 stimulation group were significantly decreased on the membrane and increased in the cytoplasm (Figure 4B).

Furthermore, both the WT and GRK5-Arg304His groups showed increased p-tau after Aβ42 stimulation, while the GRK5-Gln41Leu groups showed lower p-tau than WT groups (Figure 4C). Taken together, the results suggest that the GRK5-Gln41Leu mutant may prevent GRK5 translocation from the membrane to the cytosol as well as subsequent tau phosphorylation under Aβ42 stimulation. In contrast, the GRK5-Arg304His mutant was more sensitive to Aβ42, increasing GRK5 in the cytosol and p-tau expression.

### Analysis of AD clinical parameters

Based upon the above functional assay, we hypothesized that the two SNPs influence GRK5 function and might contribute to AD susceptibility. Thus, we further explored the relationship between the two GRK5 polymorphisms and the risk of AD in a case-control study. A total of 292 patients diagnosed with AD and 300 unrelated gender- and age-matched controls were analyzed in our study. The demographic and clinical characteristics of the study subjects are summarized in Table 1. The mean age was 73.6 years for the AD patients and 74.1 years for the control subjects; the gender (male-to-female) ratio was 1:1.41 in the case group and 1:1.31 in the control group. The distributions of age and gender did not significantly differ between the AD patients and controls (P=0.6201 and 0.4479, respectively). The MMSE score was significantly lower in AD patients than in controls (P<0.001). Moreover, as expected, the ApoEε4 allele frequencies were significantly elevated in AD patients versus those in controls (P<0.0001). All of the patient samples were genotyped for the rs2230345 and rs2230349 SNPs, and the association between the genotype and the risk of AD was analyzed using the Chi-squared test.

**Table 1:** Demographic data and clinical features of patients with AD Patients and healthy controls

Characteristics	Controls (n=300)	AD cases (n=292)	P value
Age (years)	73.6±8.29	74.1±7.72	0.4479
Gender (M/F)	130/170	121/171	0.6201
MMSE scores	28.7±0.82	18.4±6.22	<0.001
ApoEε4(+)	54	121	<0.0001

### Genetic association of AD with SNPs of GRK5 rs2230345 and rs2230349

All of the genotype distributions followed the Hardy–Weinberg equilibrium in the AD cases and controls. The allele and genotype frequencies of the GRK5 polymorphisms in the entire study population are shown in Table 2. Neither the genotype nor allele frequencies for the rs2230345 SNP showed significant differences between the AD cases and controls (genotypes P=0.223 and alleles P=0.225). However, in the LOAD subgroup, the T allele of rs2230345 SNP was associated with a lower risk of LOAD compare to the control groups (P=0.0472) (Table 2), while no differences were observed for the genotype of rs2230345 SNP (P=0.123). Moreover, the rs2230345 SNP showed no significant differences for genotype or allele frequencies in the EOAD subgroup (genotype P=0.962, allele P=0.977). With regard to the other SNP, neither the rs2230349 genotype nor the allele showed significant differences between the AD cases and the controls (genotype P=0.142, allele P=0.130) (Table 2), and no significant difference was observed in the age of disease onset subgroups (LOAD: genotypes P=0.618, alleles P=0.411, EOAD: genotypes P=0.139, alleles P=0.127) (Table 2).

**Table 2:** Frequencies of GRK5 genotypes and alleles among LOAD/EOAD subgroups in cases and controls

	Genotypes n (%)				Alleles n (%)			
rs2230345	AA	AT	TT	P-value	A	T	P-value	OR (95% CI)
AD	289 (98.97%)	3 (1.07%)	0 (0%)	0.223	581(99.49%)	3(0.51%)	0.225	2.617(0.691- 9.917)
Control	292 (97.33%)	8 (2.67%)	0 (0%)		592(98.67%)	8(1.33%)		
LOAD (>65 years)								
AD (189)	189	0	0	0.123	378	0	0.0472	8.906 (0.478-166.1)
Control (193)	189	4	0		382	4		
EOAD (≤65 years)								
AD (103)	100	3	0	0.962	206	3	0.977	0.976 (0.195-4.895)
Control (107)	104	3	0		211	3		
	**Genotypes n (%)**				**Alleles n (%)**			
**rs2230349**	**GG**	**GA**	**AA**	**P-value**	**G**	**A**	**P-value**	**OR (95% CI)**
AD	135 (46.23%)	123 (42.12%)	34(11.64%)	0.142	393 (67.3%)	191 (32.7%)	0.130	0.820 (0.640-1.051)
Control	150 (50.00%)	129 (43%)	21 (7%)		429 (71.5%)	171 (28.5%)		
LOAD (>65 years)								
AD (189)	89	80	20	0.618	258	120	0.411	0.8788(0.645-1.197)
Control (193)	96	82	15		274	112		
EOAD (≤65 years)								
AD (103)	46	43	14	0.139	135	71	0.127	0.7238(0.478-1.096)
Control (107)	54	47	6		155	59		

The individual genotype data for the two GRK5 SNPs were also analyzed for the predicted haplotypes and are shown in Table 3. For the two predicted haplotypes of the GRK5 gene (for rs2230345 and rs2230349), T-G was significantly associated with AD risk versus controls (P=0.013).

**Table 3:** The haplotype frequencies of GRK-5 polymorphisms in Chinese Han population

Haplotype rs2230345 -rs2230349	Frequency	Case Frequency	Control Frequency	P Value
A-G	0.682	0.706	0.660	0.606
A-A	0.300	0.291	0.307	0.761
T-G	0.018	0.003	0.033	**0.013**

## DISCUSSION

Mutations and SNPs of the GRK family may alter GRK function and are associated with several cardiocerebral vascular diseases, such as cardiac failure, ischemia and coronary artery disease [15–17]. GRK5 is either localized at the membrane or is recruited into the membrane to execute its primary function on G-protein-coupled receptor desensitization [18, 19]. Recent studies suggest that GRK5 deficiency may impair the desensitization of presynaptic muscarinic 2 (M2) autoreceptors [5, 20]. GRK5 deficiency may also be associated with tau hyperphosphorylation, a hallmark of AD, and play a significant role in its pathogenesis [5, 21, 22]. Our results confirm that GRK5 levels significantly increase in the membrane fraction but decrease in the cytosolic fraction of older APPsw mice. Furthermore, p396-tau expression in the hippocampus of GRK5KO mice significantly increased versus APP/PS1 transgenic mice. These results combined with previous reports further confirm that functional GRK5 deficiency causes tau hyperphosphorylation and may contribute to the development of AD [23, 24].

To assess which SNPs may affect GRK5 functionality and may be associated with AD risk, we performed a comprehensive screen of the coding region of the GRK5 gene for polymorphisms with minor allele frequency (MAF) > 0.01%. Finally, two non-synonymous polymorphisms in AD with potential functional impact were chosen to evaluate their possible effects on GRK5 function and their association with AD: cDNA nucleotide position 122 A/T changes glutamine (Gln) to leucine (Leu) at amino acid 41 (rs2230345) and nucleotide 840 G/A changes arginine (Arg) to histidine (His) at amino acid 304 (rs2230349) [25, 26].

We explored the molecular mechanism of the rs2230345 on the GRK5 function by the crystal structure. Analysis of the protein structures demonstrated that Gln41 was located in the N-terminal calmodulin/PIP2 binding domain, which is important for GRK5 binding to the membrane [27, 28]. Since leucine likely binds better to the membrane than glutamine, Gln41Leu mutation has the more ability to desensitize GPCRs [7]. CaM binding to N-terminal regions of GRK5, which largely overlap with the membrane-binding determinants, would block GRK5 association with membranes, thereby attenuating GPCR phosphorylation and leading to desensitization of the receptors [29–32]. The substitution of the polar amino acid Gln41 with the non-polar amino acid Leu41 might be responsible for promoting the stability of GRK5 on the membrane, thereby strengthening receptor phosphorylation activity, which is consistent with reports involving heart failure [18, 29]. As our results indicated, there were no obvious changes in the distribution of GRK5 in the GRK5-Gln41Leu group. However, the Gln41Leu mutation did inhibit GRK5 translocation from membrane to cytoplasm and protect tau hyperphosphorylation in SH-SY5Y cells after Aβ42 stimulation. As might be expected for a polymorphism within a putative regulatory domain, Leu41 was markedly more effective than Gln41 in promoting the stability of GRK5 on the membrane and inhibiting tau hyperphosphorylation when cells were stimulated with Aβ42. Consistent with the findings obtained from the functional assay and case-control study, the missense variation rs2230345 in the GRK5 gene is associated with LOAD resistance by its T allele distribution. The GRK5-Gln41Leu (rs2230345) mutant could influence the translocation of GRK5 and may protect the tau hyperphosphorylation in the pathology of AD, which ultimately may contribute to LOAD susceptibility. Taken together, the results of our study indicate that GRK5-Gln41Leu might serve as a potential marker for LOAD resistance.

In contrast, the Arg304His mutation affects the function of the GRK5 catalytic domain rather than the membrane-binding function and was more sensitive to Aβ42, leading to increased levels of GRK5 in the cytosol and the expression of p-tau [28]. Arg304 was located in the C-domain, which might interact with the kinase domain. Studies have shown that when arginine 304 was exchanged for cysteine the kinase appeared to be evenly distributed between the cytosol and the plasma membrane. The substitution of Arg-to-His at amino acid 304 of GRK5 might disrupt the catalytic functions of the kinase domain. As our results indicate, when arginine 304 was exchanged for histidine, it induced an increase in GRK5 cytoplasm and tau hyperphosphorylation. We hypothesized that the gain-of-function Gln41Leu mutation in GRK5 enhances its membrane-binding properties, thereby attenuating receptor desensitization, which has protective effects for AD. However, the Arg-to-His substitution at amino acid 304 in GRK5 is a loss-of-function mutation that might disrupt the catalytic functions of the kinase domain, enhancing the toxicity of Aβ42.

GRK5 encodes a member of the guanine nucleotide-binding protein (G protein)-coupled receptor kinase subfamily of the Ser/Thr protein kinase family and is located in chromosome band 10q26.11 [32–35]. GRK5 dysfunction affects muscarinic signaling and promotes tau hyperphosphorylation through PKC-mediated activation of GSK3β signaling pathways *in vivo*, which might be a potential pathogenic link in AD [23]. GRK5 deficiency appears to connect cholinergic hypofunctioning and Aβ42 accumulation in a self-promoting loop cycle that accelerates both activities [9, 36]. As described, GRK5 deficiency has emerged to become a critical player in AD pathogenesis that might result from excess Aβ42 accumulation and that can conversely enhance Aβ42 toxicity, leading to cholinergic dysfunction and tau hyperphosphorylation.

**Figure 5:**
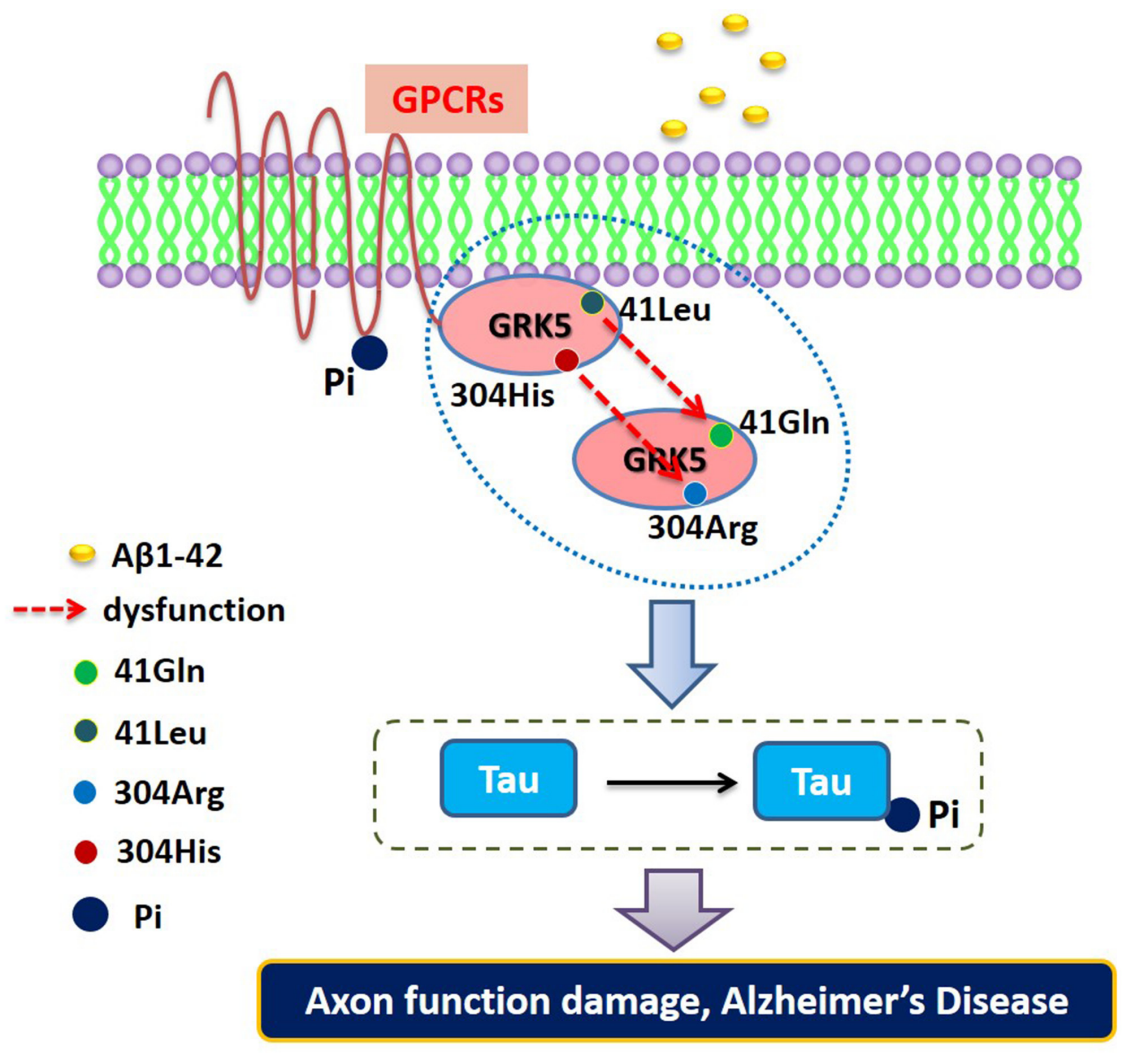
The functional mapping for evaluating the impact of the rs2230345 (GRK5-Gln41Leu) and rs2230349 (GRK5-Arg304His) polymorphisms on GRK5 function and tau phosphorylation

Based on previous studies, we propose a functional mapping for evaluating the impact of the rs2230345 and rs2230349 polymorphisms on GRK5 function and tau phosphorylation. As shown in Figure 5, GRK5-Gln41Leu performed a gain-of-function by enhancing its membrane-binding properties, which serves as a resistance in AD patients. However, the GRK5-Arg304His mutant might function as GRK5 deficiency *in vivo*, which was more sensible to Aβ. In this vicious cycle, Aβ42 and cholinergic dysfunction each serve as the causes and consequences, whereas GRK5 deficiency amplifies these effects [37]. In addition, it has been reported that the GRK5 dysfunction constitutes a crucial regulatory step for tau hyperphosphorylation by activating GSK3β signaling pathways, another known risk factor for AD [23, 38].

Some potential limitations of our study should be addressed. First, we found that two SNPs affected GRK5 function, but only rs2230345 showed a significant correlation in the case-control study. We speculate that the results of the case-control analysis in AD remain preliminary due to the small number of subjects, and further investigation of the GRK5 polymorphisms with a larger and more ethnically diverse population of AD patients is warranted. In addition, these results are based primarily on *in vitro* cell culture models. We will use transgenic animal models for *in vivo* studies aimed to explore the association between the two polymorphisms and the pathology process of AD.

In conclusion, our results suggest that the two SNPs, rs2230345 and rs2230349, may contribute to AD susceptibility through their influence on GRK5 function and tau hyperphosphorylation. Therefore, these functional genetic variants of GRK5 merit consideration and further investigation as therapeutic targets for the treatment of AD, offering opportunities for novel personalized medicine approaches.

## MATERIALS AND METHODS

### Animal preparation

Three murine models were used in this study, GRK5 knockout (GRK5KO) mice (GRK5^+/−^), APP/PS1 double transgenic (AD) mice (APP^+/−^/PS1^+/−^) and wild-type (WT) C57BL/6 mice (GRK5^+/+^/APP^−/−^/PS1^−/−^). AD and WT mice were acquired from the Institute of Laboratory Animal Center of Guangdong Province (Guangdong, China). GRK5KO mice were acquired from Cyagen Biosciences (Guangzhou) Inc. (Guangzhou, China). The GRK5 gene was targeted with the Cas9 protein using a guide-miRNA in zygote cells. The targeting structure introduced two guide-miRNA matching sites in the transcript flanking exons 2 and 3 as well as with a marker gene cassette near the matching sites. Microinjection of homologous embryonic stem cell (ESC) clones with Cas9-recombinase resulted in the loss of the sequences between the two matching guide-miRNA sites, leading to deletion of the marker genes and exons 2 and 3 of the GRK5 gene. When GRK5 mutant ESCs divided into blastocysts, embryo transplantation was performed in C57BL/6 mice. Three genotypes were produced from these mice: GRK5 mutant heterozygote mice (GRK5^+/−^), GRK5 mutant homozygous mice (GRK5^−/−^) and wild-type GRK5 mice. PCR amplification and DNA genotyping were used to identify knockouts. A homozygous GRK5KO strain (GRK5^−/−^) was generated by cross breeding GRK5^+/−^mice together for at least 6 generations. All mice were housed in a specific-pathogen-free (SPF) room with normal daily periodicity and were provided with sterile rodent chow and water at regular intervals.

The use of these animals was approved by the Guangdong Medical University Laboratory Animal Center Committee, and the experiments were performed according to the permitted agreements. Experimental animals were conventionally raised a week after purchase with a room temperature maintained at 22-25°C and 10-50% relative humidity with a 12 h light/dark cycle. The animals were allowed free access to food and water.

### Preparation of human β-Amyloid 1-42 (Aβ42) oligomers

Human Aβ42 was purchased from ChinaPeptides Co., Ltd. (Shanghai, China). Aβ42 was dissolved at a final concentration of 1 mg/ml with the addition of hexafluoroisopropanol (HFIP) followed by a 10 min ultrasound bath, vacuum drying and redissolution. Eventually, an Aβ42 solution (Aβ42 monomers) was dissolved in dimethyl sulfoxide (DMSO) at a final concentration of 2 mg/ml. The fresh Aβ42 solutions were used for experiments immediately, and the remaining solution was stored at −80°C.

### DNA isolation and genotyping for the SNPs

Peripheral blood samples were collected, and genomic DNA was isolated using the blood Genomic DNA Extraction Kit (Tiangemn, China). The genomic DNA was genotyped using the ABI PRISM SNapShot method (Applied Biosystems, Foster, CA, USA). Two target fragments were captured by amplification with two pairs of primers that included rs2230345-F: 5′-GAGCCTCAGCCAGGCATCTTTT-3′, rs2230345-R: 5′-CGGCTCACCCATTTGCCTTAAT-3′, rs2230349-F: 5′-TCAACCTGGCCTATGCCTACGA-3′ and rs2230349-R: 5′-CTTGGAAGTCCTTGGTGCCTTCC-3′. Briefly, a 10 μl final volume of PCR reaction mixtures was added to 5 μl SNapShot Multiplex Ready Mix, 1 μl primers and 4 μl DNA templates that were purified with the QIAquick PCR Purification Kit (QIAGEN, Hilden, Germany). The PCR cycling program was as follows: an initial cycle at 95°C/2 min; 25 cycles of 94°C/30 s, 57°C/30 s and 72°C/40 s; and a final cycle at 4°C until analysis. The extension products were then purified with 1 U of shrimp alkaline phosphatase (SAP) and exonuclease I at 37°C for 15 min followed by a 15 min incubation at 80°C to inactivate the enzyme. After purification with SAP, the extension products were analyzed with the ABI 3730xl DNA Analyzer and GeneMapper 4.1 (Applied Biosystems, Carlsbad, CA, USA). At last, 5% of the random duplicate samples from each genotyping were selected for quality control.

### 3D structure modeling and GRK5 docking study

The crystal structures of the GRK5 (PDB ID: 4WNK, Homan KT et al., 2015) were established using the Insight II/Builder program [39]. The structure of SNP-encoding GRK5 proteins was predicted based on the native conformation to determine how the Gln41Leu or Arg304His amino acid changes encoded by the SNPs altered protein structure. The analysis was visualized using SWISSPDB viewer. The root mean square deviation values were calculated to correct for the structural deviation between the native and mutant GRK5 proteins. The structural integrity of the models was visually examined with Discovery Studio (DS) Visualizer 3.1 (http://accelrys.com/products/discoverystudio/ visualization-download.php), which was also used to generate images of the structures.

### Plasmid construction and transfection

The DNA fragment encoding the GRK5 precursor was ligated to the pre-cut GV230 vector, and an enhanced green fluorescent protein (eGFP) tag was added to the GRK5 C-terminal. Two GRK5 mutant plasmids (rs2230345 and rs2230349) and a wild-type (wt) GRK5 plasmid were constructed. The polymorphism at rs2230345 was amplified with the primers 5′-ACATTAGCCTGTGT GAAGACCTCCGAAGGACCATAG-3′ and 5′-GTCTTCACACAGGCTAATGTGAGGGAACTTCAG-3′. For rs2230349, the polymorphism was amplified with the primers 5′-ACCTCCACCATGAGAACACCGTCTACCGAGATC-3′ and 5′-GGTGTTCTCATGGTGGAGGTCTTCTAAGCCGCAG-3′. Site-directed mutagenesis was performed to modify single bases. In the rs2230345 plasmid, the base 188 (A) was mutated to T and in the rs2230349 plasmid, the base 975 (G) was mutated to A. The PCR products from these three plasmids were the same size, and their sequences were exactly the same except for the mutation site. All of the plasmid constructs were generated and authenticated by Genechem Inc. (Shanghai, China). Before transfection, SH-SY5H cells were seeded in six-well plates at approximately 70% confluence in complete medium (DMEM supplemented with 10% FBS) without antibiotics. Opti-medium (100 μl) mixed with 1 μg plasmid DNA and 1 μl DNA-IN SY5Y transfection reagents (GlobalStem, USA) constituted the transfection complex, and a volume of 62.5 μl were added to each well before incubation at 37°C in 5% CO_2_ for 6 h. After incubation, plasmid enriched medium was replaced with routine medium and the cells were cultured for 24-48 h under the same condition. The expression of green fluorescence was observed with a fluorescence microscope (Leica DMI300B, Germany) and was used to calculate the transfection efficiency.

### Cell culture and Aβ42 administration

SH-SY5Y cells purchased from Genechem Inc. (Shanghai, China) were cultured in complete Dulbecco's Modified Eagle's Medium (DMEM) supplemented with 10% fetal bovine serum (FBS) and 1% penicillin- streptomycin antibiotics at 37°C in 5% CO_2_. Confluent cell cultures were passaged with 0.25% trypsin containing EDTA as previously described [40]. For the best results, continuous passages for at least two generations were recommended to restore cell activity after thawing. A liquor stock solution of Aβ42 was prepared by gently mixing 113 μl Aβ42 solutions (2 mg/ml) and 887 μl PBS (1 mM). The dilute solutions of Aβ42 (50 μM) were incubated in a water bath for 30 min at 37°C to activate monomers and form the Aβ42 aggregates. In our study, final concentrations of 5 μM and 1 μM Aβ42 aggregates were used to form the concentration gradient, and Aβ42-treated cells were cultured at 37°C in 5% CO_2_ for 24 h before subsequent experiments.

### Western blotting analysis

Transfected cells or non-transfected cells at 90% confluence were harvested and lysed in RIPA buffer (Solarbio, Beijing, China) complemented with 2% protease inhibitor cocktail (Roche Applied Science, Mannheim, Germany), 1% PMSF (Solarbio, Beijing, China) and 1% NaF (Solarbio, Beijing, China). In addition to total protein extraction, membrane proteins and cytosolic proteins were extracted using the Membrane Protein Extraction Kit (Thermo Fisher, USA), and the procedures were performed in accordance with the manufacturer's protocols. Three types of transfected cells were used to detect membrane and cytosolic proteins in cells robustly expressing wild-type GRK5, rs2230345 or rs2230349. For the mice, the hippocampus on both sides was detached from the deep cortical layer and homogenized with RIPA complexes and cell lysis solution. The following steps were performed similarly regardless of the source of the protein (cells or tissues) as previously described [41]. The only differences were the antibody used and their dilution ratios, which included phosphorylated tau, or p-tau, (Abcam; 1:1000), tau (Cell Signaling; 1000), GRK5 (Abcam; 1:600) and beta-actin (Solarbio; 1:1000).

### Patient samples

Samples from 292 AD patients and 300 matched individuals were obtained from the Shenzhen Nanshan Center for Disease Control and Prevention between April 2008 and February 2013. The clinical diagnosis of AD was based upon the revised criteria of the National Institute of Aging and Alzheimer's Association (NIA-AA). The Mini-Mental Status Examination (MMSE) was used to score cognitive impairment as previously described [40]. The AD group samples were confirmed to come from patients who did not have other types or compound types of dementia. The control group samples were confirmed to come from patients who were free of nervous system diseases and a variety of acute illnesses, including hyperpyrexia and acute coronary syndrome. Samples from patients in the active stages of other diseases were excluded from both groups. All experiments on human subjects were conducted in accordance with the Declaration of Helsinki. Written informed consent was obtained from all the enrolled participants, and this study was approved by the Ethics Committee of the Affiliated Hospital of Guangdong Medical University.

### Statistical analyses

The data were presented as the means ± SEM, and variance analysis was performed using SPSS 19.0. Genotype and allele frequencies were quantified. The Hardy–Weinberg equilibrium was estimated between the expected and actual genotype distributions using Chi-squared test. The student's t-test was used for genotypic distributions and enumeration data (allele and genotype distributions), which were compared using Chi-squared test or Fisher's exact. In the sub-categories of age and ApoEε4 carrier, analyses were repeated as above. The odds ratios (OR) represented the strength of the correlation, and the 95% confidence interval (CI) was used to evaluate prevalence risks. Haplotype analysis was performed using Haploview 4.2. In all comparisons, P values were calculated and corrected for the assessment of statistical significance; P<0.05 was considered statistically significant.

## Abbreviations

Aβ: β-amyloid
AD: Alzheimer's disease
ApoE: apolipoprotein E
APP: amyloid precursor
EOAD: Early Onset Alzheimer Disease
ELISA: enzyme-linked immunosorbent assay
ESC: embryonic stem cell
FBS: fetal bovine serum
GPCRs: G protein-coupled receptors
GRK5: G protein-coupled receptor kinase-5
LOAD: Late Onset Alzheimer Disease
PCR: polymerase chain reaction
PSEN1: presenilin 1
PSEN2: presenilin 2
SNPs: single-nucleotide polymorphisms
SPF: specific pathogen free
